# Generating cerebellar organoids from pluripotent stem cells

**DOI:** 10.1242/dmm.052478

**Published:** 2026-01-30

**Authors:** Esther B. E. Becker, Simone Mayer, Lena M. Kutscher

**Affiliations:** ^1^ Nuffield Department of Clinical Neurosciences, University of Oxford, Oxford OX3 9DU, UK; ^2^ Kavli Institute for Nanoscience Discovery, University of Oxford, Oxford OX1 3QU, UK; ^3^ Karlsruhe Institute of Technology, Zoological Institute, 76131 Karlsruhe, Germany; ^4^ Karlsruhe Institute of Technology, Institute of Biological and Chemical Systems - Functional Molecular Systems (IBCS-FMS), 76131 Karlsruhe, Germany; ^5^ Developmental Origins of Pediatric Cancer Junior Research Group, Hopp Children's Cancer Center (KiTZ) and German Cancer Research Center (DKFZ), 69120 Heidelberg, Germany

**Keywords:** Cerebellum, Development, Organoid, Disease models, Quality control

## Abstract

Cerebellar organoids present promising tools for the modelling of human cerebellar development and diseases. As this young field grows, robust standards and transparent reporting practices are needed to ensure the reproducibility and utility of the generated cerebellar organoid models. Here, we summarize current approaches to generate cerebellar organoids and their applications. We suggest common quality control standards and biological readouts that should be considered in this emerging area.

## Teaser

**Summary:** This Special Article provides a comprehensive overview of the methods, applications and considerations for the generation of cerebellar organoids.

## Introduction

The cerebellum is one of the first brain structures to develop but one of the last to mature. In humans, the cerebellum starts to develop early during the first trimester (∼30 days post-conception) (reviewed in Haldipur et al., 2022) and continues to grow throughout childhood and adolescence, reaching its peak volume between the ages of 12 and 16 years (Tiemeier et al., 2010). Its protracted development makes the cerebellum particularly vulnerable to genetic and environmental insults, which can manifest as structural malformations, neurodevelopmental diseases including autism spectrum disorder, and childhood brain tumours, such as medulloblastoma. Cerebellar developmental mechanisms are conserved between species, and much has been learned about how the cerebellum forms from studies in model organisms such as mouse (reviewed in Leto et al., 2016). However, recent studies have highlighted multiple aspects unique to the development of the human cerebellum, such as the expanded size of the human cerebellum (Haldipur et al., 2019), the relative abundance and gene expression programmes of specific cell types (Sepp et al., 2024), and the presence of unique developmental structures (Erickson et al., 2025; Haldipur et al., 2019), as discussed in further detail below.

One approach to bridging the gap between animal and human studies is the development of human-specific, stem cell-derived *in vitro* models. Neural organoids are three-dimensional (3D), self-organized structures derived from pluripotent stem cells that offer a unique tool to model and investigate previously inaccessible aspects of early human brain development (reviewed in Eichmüller and Knoblich, 2022; Birtele et al., 2025) and diseases associated with it. Over the past decade, our understanding of mechanisms underlying early brain development has progressed, enabling the establishment of protocols tailored to the differentiation of cerebellar organoids from human pluripotent stem cells (Atamian et al., 2024; Muguruma et al., 2010; Nayler et al., 2021; Silva et al., 2020). There is growing interest in using cerebellar organoids to model development and evolution, and to understand disease mechanisms that underlie disorders affecting the human cerebellum. As this field grows, the reproducibility and translatability of the generated cerebellar organoid models are imperative. A general framework for neural organoid models has recently been proposed, which includes ensuring that the model system is capable of answering the specific scientific question, transparency in experimental details, and high quality of the human pluripotent stem cells as a foundational step (Paşca et al., 2025). Advancements in using brain organoids from other brain regions to model disease and development have also been summarized in other reviews (Birtele et al., 2025; Lancaster and Huch, 2019). The emerging field of cerebellar organoids would benefit from similar guidelines for experimental design and reporting, ensuring the reproducibility and translatability of findings. Therefore, we have developed practical guidance for the generation of cerebellar organoids to support researchers interested in starting experiments, based on published literature and the authors' shared experience. This guidance could also promote common standards and aid in advancing this field of research.

## Development of cerebellar organoids

The experimental steps to generate cerebellar organoids are guided by the mechanisms underlying normal cerebellar development.

### Cerebellar development

**Fig. 1.**
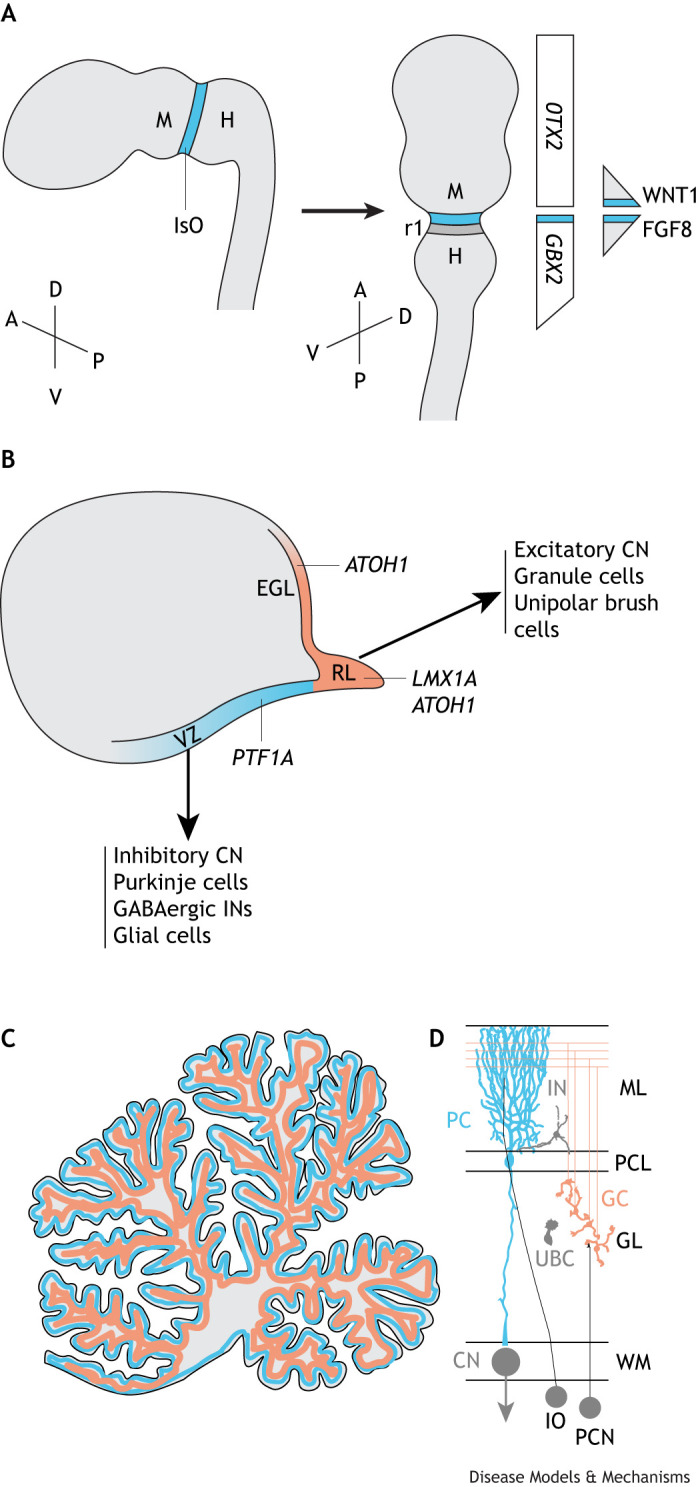
**Key features of human cerebellar patterning and gene expression.** (A) The midbrain (M)–hindbrain (H) boundary is set shortly after neural tube closure at ∼4-5 post-conception weeks (PCW). The isthmic organizer (IsO) secretes the morphogens WNT1 and FGF8 to set the boundary, which establishes a gradient of decreasing concentration (triangle shapes on the right of the figure). Rhombomere 1 (r1) of the hindbrain, which will generate the cerebellum, expresses the transcription factor *GBX2* (expression throughout the hindbrain, which then tapers out at the spinal cord) but not the forebrain-associated transcription factor *OTX2* (expression relatively constant in the developing forebrain and midbrain). The developing brain is shown along two orientations of the anterior (A) –posterior (P) and dorsal (D)–ventral (V) axes. (B) Later in development, around PCW 10, two progenitor pools are specified, including the ventricular zone (VZ; blue), which expresses *PTF1A*, to generate all inhibitory neurons and glial cells, and the rhombic lip (RL; orange), which expresses first *ATOH1* and then *LMX1A*, to generate all excitatory neurons. The external granule layer (EGL), expressing *ATOH1*, is a second germinal zone producing the abundant excitatory granule neurons of the cerebellum. (C) At later stages in development, the cerebellum becomes highly foliated and adopts its characteristic three-layer structure, represented here in grey [molecular layer (ML)], blue [Purkinje cell layer (PCL)] and orange [granular layer (GL)]. (D) Major cell types of the mature cerebellum include Purkinje cells (PC; blue), granule cells (GC; orange), unipolar brush cells (UBC; grey), GABAergic interneurons (IN; grey) and cerebellar nuclei (CN; grey). Note that not all cell types of the cerebellum are shown. Climbing fibre inputs from the inferior olive (IO) and mossy fibre inputs from the precerebellar nuclei (PCN) are not modelled in cerebellar organoids, but could potentially be modelled by assembloids in the future. *ATOH1*, atonal bHLH transcription factor 1; FGF8, fibroblast growth factor 8; *GBX2*, gastrulation brain homeobox 2; *LMX1A*, LIM homeobox transcription factor 1 alpha; *OTX2*, orthodenticle homeobox 2; *PTF1A*, pancreas associated transcription factor 1a; WM, white matter.

During embryonic development, the region (anlage) in the developing neural tube that will give rise to the cerebellum is generated through signalling from a part of the neuroepithelium known as the isthmic organizer (IsO) at the midbrain–hindbrain boundary (Fig. 1A). This region is demarcated by the coordinated expression of patterning genes, including orthodenticle homeobox 2 (*OTX2*), gastrulation brain homeobox 2 (*GBX2*), engrailed homeobox (*EN*)*1*, *EN2* and paired box 2 (*PAX2*) (reviewed in Butts et al., 2014; Leto et al., 2016). The IsO acts as a key orchestrator of cerebellar induction via secretion of fibroblast growth factor (FGF)8, which works in concert with other morphogens such as WNT1, sonic hedgehog (SHH) and transforming growth factor beta (TGF-β) family members (reviewed in Leto et al., 2016). Secreted FGF8 and WNT1, along with other transcription factors, form a positive feedback loop driving each other's expression that supports the ability of the IsO to self-induce, maintain itself and guide tissue patterning (Muguruma et al., 2010). Following the territorial specification of the cerebellar anlage, two germinal centres form the ventricular zone (VZ) and the rhombic lip (RL) (Fig. 1B), which give rise to all cerebellar neurons in successive waves. The VZ, marked by the expression of the basic helix-loop-helix (bHLH) transcription factor pancreas associated transcription factor 1a (*PTF1A*) gives rise to all cerebellar GABAergic neuron cell types: first inhibitory cerebellar nuclei (CN) neurons, then Purkinje cells and, finally, GABAergic interneurons (Fig. 1B; Hoshino et al., 2005). After the completion of the neurogenic phase, progenitors in the VZ undergo a gliogenic switch, giving rise to glial precursors that ultimately differentiate into cerebellar astrocytes (Buffo and Rossi, 2013). The RL is marked by expression of another bHLH transcription factor, atonal bHLH transcription factor 1 (*ATOH1*), and generates progenitor cells that give rise to all cerebellar glutamatergic neurons, starting with large excitatory CN neurons, followed by granule cells and then unipolar brush cells (Fig. 1B; Machold and Fishell, 2005). The subsequent expansion and migration of cerebellar progenitors result in the characteristic three-layered structure of the cerebellar cortex with its unique foliation pattern and the formation of the cerebellar nuclei (Fig. 1C).

### Human-specific aspects of cerebellar development

The above-described processes that shape the developing cerebellum are highly conserved in vertebrates (reviewed in Butts et al., 2014; Lowenstein et al., 2023). However, important differences between species exist that influence cerebellar architecture and function, with implications for disease. Compared to cerebellum in other species, the human cerebellum has a significantly larger surface area (∼750-fold greater than that of mouse cerebellum and tenfold greater than that of non-human primate cerebellum) (Van Essen, 2002), has greatly enlarged hemispheres and is extensively foliated (Altman and Bayer, 1997). The human cerebellum encompasses 80% of all brain neurons, i.e. a total of ∼69 billion neurons, compared to 60% in mouse (∼10 billion cerebellar neurons) (Herculano-Houzel, 2010). Moreover, neuronal subtype ratios differ between species, with a much higher granule cell-to-Purkinje cell ratio in human (3000:1) than in mouse (200:1) (Lange, 1975). In addition, the human cerebellum has a twofold higher peak percentage of Purkinje cells early during development than mouse cerebellum (Apsley and Becker, 2022), followed by a selective expansion of early-born Purkinje cell subtypes that is unique to humans (Sepp et al., 2024). Many of these differences are likely due to the distinctive generation of GABAergic (Erickson et al., 2025) and glutamatergic progenitors (Haldipur et al., 2019) in the human cerebellum, compared to that in other species; during the protracted development of the human cerebellum, the VZ continues to expand for longer and is split into a VZ and a subventricular zone (SVZ), reminiscent of the subdivision of the VZ in the developing cerebral cortex (Haldipur et al., 2019). Similarly, the human RL is both spatiotemporally expanded and compartmentalized and splits into two molecularly distinct substructures, the RL-VZ and RL-SVZ, which are proposed to be separated by a vascular bed (Haldipur et al., 2019). Uniquely, the human RL becomes embedded within the posterior lobule of the cerebellum after mid-gestation, where it remains proliferative until birth (Haldipur et al., 2019). In addition to likely contributing to the overall increased neuronal number, surface area and complexity of the human cerebellum, these developmental differences are also relevant to neurodevelopmental disorders. For example, disruption of RL development has been linked with the rare congenital brain condition Dandy-Walker malformation (Haldipur et al., 2021) and the formation of the paediatric brain cancer medulloblastoma (Hendrikse et al., 2022; Okonechnikov et al., 2023; Smith et al., 2022). The role of the specific RL compartments in these diseases requires additional investigation.

### Generation of human cerebellar organoids from pluripotent stem cells

**Fig. 2.**
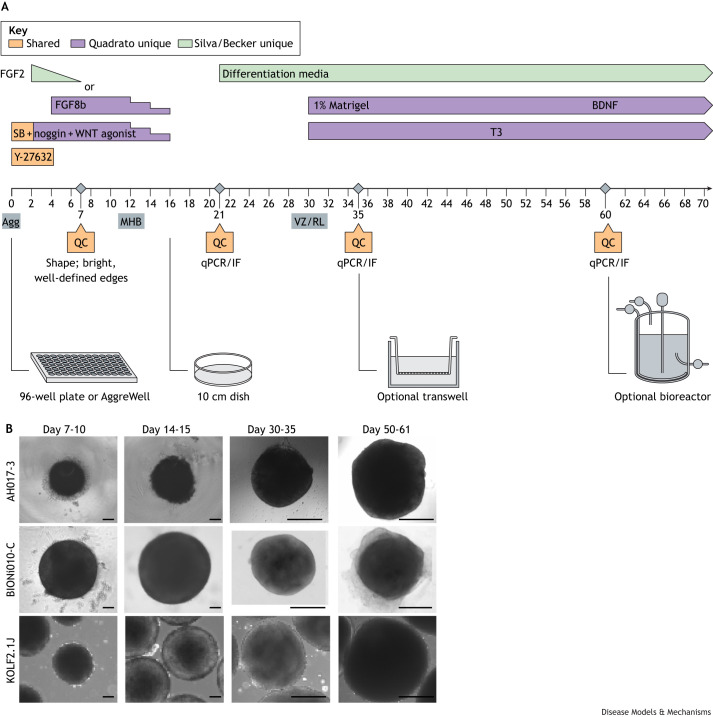
**Key points of quality control measures for cerebellar organoid differentiation.** (A) All four published differentiation protocols share some features for differentiation (orange). The key difference is the use of FGF2 (Silva, Becker, Muguruma protocols; green) versus FGF8 (Quadrato protocol; purple). When differentiating organoids for the first time or when using a new induced pluripotent stem cell (iPSC) line, it is essential to perform quality control (QC) experiments at days 7, 21, 35 and 60. Organoids can be grown optionally in transwells or bioreactors at later stages. Agg, aggregates; BDNF, brain-derived neurotrophic factor; FGF, fibroblast growth factor; IF, immunofluorescence; MHB, midbrain–hindbrain boundary; qPCR, quantitative PCR; RL, rhombic lip; SB, SB431542; T3, triiodothyronine; VZ, ventricular zone. (B) Example organoids using different iPSC lines in three different laboratories: AH017-3 iPSCs differentiated using FGF2 protocol (Nayler et al., 2021) (top row); BIONi010-C (middle row) and KOLF2.1J (bottom row) iPSCs differentiated using FGF8 protocol (Atamian et al., 2024). Scale bars: 100 µm (first two columns) and 500 µm (last two columns). Organoid images courtesy of Tamiris Borges de Silva (University of Oxford), Theresa Kagermeier (Karlsruhe Institute of Technology) and Frederik Arnskötter (DKFZ).

Protocols for generating human cerebellar organoids aim to recapitulate cerebellar development *in vitro* by relying on knowledge of the *in vivo* morphogens that pattern the cerebellum. Building on their earlier work using mouse pluripotent stem cells (Muguruma et al., 2010), Muguruma and colleagues demonstrated that aggregated human embryonic stem cells form an IsO-like tissue in response to TGF-β inhibition with SB431542, which promotes the formation of neuroectoderm (neuralization), and treatment with insulin and FGF2, which induces a hindbrain fate (Muguruma et al., 2015) (Fig. 2A). Under these conditions, organoids showed expression of midbrain–hindbrain markers *GBX2* and *EN2*, and the IsO-organizing morphogens *FGF8* and *WNT1*, thus recapitulating the endogenous self-inductive signalling events of early cerebellar specification. This induction was followed by the expression of markers of kin of IRRE-like protein 2 (KIRREL2)- and PTF1A-positive VZ and ATOH1-positive RL progenitors. The addition of the growth factor FGF19, which is critical for cell proliferation and survival in the developing cerebellum (Miyake et al., 2005), promoted the formation of larger, polarized neuroepithelial structures with KIRREL2-positive Purkinje cell progenitors on the outside (Muguruma et al., 2015). Additional treatment with stromal cell-derived factor 1 (SDF-1), a chemoattractant that is secreted from the meninges in the developing cerebellum (Klein et al., 2001), helped to stratify the neuroepithelium into VZ- and RL-like zones, which were reminiscent of the layered cytoarchitecture of the developing cerebellum.

Subsequent studies have adapted the described above initial protocol for the differentiation of human induced pluripotent stem cells (iPSCs) into cerebellar organoids (Silva et al., 2020; Watson et al., 2018), followed by long-term maintenance using bioreactors (Silva et al., 2021), suspension culture (Chen et al., 2023) or air–liquid interface culture on transwells for their long-term maintenance (Nayler et al., 2021). Supporting the key role of FGF2 in the cerebellar patterning of iPSC-derived brain organoids, a recent multiplexed morphogen screen of 14 morphogen modulators identified FGF2 as the dominant morphogen driving efficient generation of cerebellar neurons from pluripotent stem cells (Amin et al., 2024).

An alternative approach for the differentiation of cerebellar organoids from human pluripotent stem cells has used dual SMAD inhibition (SB431542/noggin) for neuralization, followed by activation of WNT signalling (CHIR-99021) and treatment with FGF8b to mimic IsO signalling *in vitro* (Atamian et al., 2024) (Fig. 2A, Table 1). Similarly to the protocol described above, this protocol also yielded robust expression of midbrain–hindbrain markers, followed by the generation of VZ- and RL-derived progenitors and maturing cerebellar neural cell types during long-term culture in suspension under shaking or bioreactor conditions. The addition of SDF-1 within this protocol also led to the formation of distinct VZ- and RL-like zones. However, using a lower SDF-1 concentration for a longer time period resulted in a reversed polarity (Atamian et al., 2024) compared with that in earlier studies (Muguruma et al., 2015), with BARHL1-positive RL-derived cells on the outside of the organoid and calbindin-positive VZ-derived cells on the inside (Atamian et al., 2024), thereby more closely mimicking the architecture of the developing cerebellum. To note, the SDF-1-induced layered organoid structure was not maintained during long-term culture after SDF-1 treatment was stopped (Atamian et al., 2024).

**Table 1.** Comparison of current differentiation methods

Differentiation protocol	Aggregate formation	Patterning molecules	PSC lines used	Additional studies using this method
Muguruma et al. (2015)	96-well V-bottom plate	SB431542, insulin, FGF2	H9 ESC	Medulloblastoma modelling (Ballabio et al., 2020)
Silva et al. (2020)	AggreWell	SB431542, insulin, FGF2	F002.1A.13, iPSC6.2	PCH modelling (Kagermeier et al., 2024)
Nayler et al. (2021)	96-well V-bottom plate	SB431542, insulin, FGF2	WTC-11, AH017-3	Medulloblastoma modelling (van Essen et al., 2024)
Atamian et al. (2024)	96-well V-bottom plate	SB431542/noggin, CHIR-99021, FGF8b	PGP1, 11a, D2	Conference report (Kutscher et al., 2025)

ESC, embryonic stem cell; FGF, fibroblast growth factor; PCH, pontocerebellar hypoplasia; PSC, pluripotent stem cell.

These two protocols (Atamian et al., 2024; Muguruma et al., 2015) used different cell lines, different media compositions, and different concentrations and durations of SDF-1 treatment, making a direct comparison difficult (Table 1, Fig. 2A). However, the findings suggest that, in contrast to in cerebral organoids (Lancaster et al., 2013), laminar layering may not be intrinsically encoded in cerebellar organoids and highlights the need for well-timed and -dosed administration of external cues to recapitulate the cytoarchitecture of the developing cerebellum *in vitro*. Single-cell RNA sequencing (scRNA-seq) of cerebellar organoids demonstrated that both FGF2- and FGF8-driven protocols generate major cell types of the developing human cerebellum including both RL- and VZ-derived neurons (Amin et al., 2024; Atamian et al., 2024; Chen et al., 2023; Nayler et al., 2021; Sarieva et al., 2024 preprint), underscoring the potential of these *in vitro* methods for recapitulating neurogenesis during human cerebellar development.

### Modelling of neurodevelopmental diseases

In recent years, several studies explored the potential of cerebellar organoids to model neurodevelopmental disorders. Studies by Atamian et al. (2024) and Nayler et al. (2021) suggest that cerebellar organoids may serve as an *in vitro* model for medulloblastoma, as scRNA-seq has identified RL-derived progenitor cells that are among the cells-of-origin for this paediatric brain cancer. Medulloblastoma is a highly heterogeneous tumour type, composed of four molecular subtypes (WNT, SHH, Group 3 and Group 4), which have distinct molecular drivers, cells-of-origin and clinical outcomes (Northcott et al., 2019). Two different approaches have been pursued for the modelling of medulloblastoma (Ballabio et al., 2020; van Essen et al., 2024), which differ primarily through their distinct methodological strategies, although they also target different subtypes. In the first approach, somatic mutations are modelled through the introduction of oncogenes after cerebellar organoids have formed, such that only some cells within the organoid carry the aberration. The second approach better examines germline predisposition genes through mutations at the level of iPSCs, such that all cells within the organoid carry the genetic change.

To model Group 3 medulloblastoma, Ballabio et al. (2020) employed the first approach and overexpressed oncogenic drivers in cerebellar organoids at day 35 of differentiation by electroporation, resulting in overproliferation of SOX9-positive progenitor cells, and brain tumour formation upon intracranial injection into immune-compromised nude mice. To model SHH-subtype medulloblastoma, van Essen et al. (2024) employed the second approach and used clustered regularly interspaced short palindromic repeats (CRISPR)-based gene editing to introduce characteristic loss-of-function germline mutations in patched 1 (*PTCH1*). Cerebellar organoids differentiated from *PTCH1-*heterozygous iPSCs contained expanded RL and granule cell progenitor populations and displayed features associated with preneoplastic stages of medulloblastoma (van Essen et al., 2024).

These studies demonstrate the potential of cerebellar organoids to model tumour initiation; however, the lack of immune and vascular components in current differentiation protocols means that they do not adequately recapitulate the tumour microenvironment. To date, cerebellar organoids have only modelled some subtypes of medulloblastoma, and current mouse models also adequately model patient tumours by overexpression of the oncogene *Myc* and deletion of the tumour-suppressor gene *Ptch1*. Nonetheless, cerebellar organoids have the potential to model aspects of tumour formation not possible in animal models, including the modulation of the human genome structure, the examination of the influence of human-specific genes and the examination of cellular changes in a human cellular context. Hence, they may become promising models to test drugs and model patient-specific features in line with personalized medicine approaches.

Cerebellar organoid modelling has also been used for the study of pontocerebellar hypoplasia (PCH), a congenital neurodegenerative disorder of the cerebellum (Kagermeier et al., 2024). The main subtype of PCH, PCH2a, is caused by a missense variant in tRNA splicing endonuclease subunit (TSEN54) (p. TSEN54 A307S) (Budde et al., 2008). *Tsen54* knockout mouse models do not accurately model PCH, as loss-of-function of the entire gene leads to major developmental disturbances that do not mirror the patient phenotype (Ermakova et al., 2018; Kasher et al., 2011; Schmidt et al., 2022). At the same time, biochemical studies using overexpression of the TSEN complex in heterologous cells (Hayne et al., 2023; Sekulovski et al., 2023; Yuan et al., 2023) and analysis of TSEN complex activity in human patient fibroblasts (Sekulovski et al., 2021) have only shown subtle biochemical changes that could not be attributed to the severe clinical phenotype. By contrast, patient-derived iPSCs, harbouring the missense variant p.TSEN54 A307S, mimicked the PCH patient phenotype when differentiated into cerebellar organoids (Kagermeier et al., 2024). The PCH organoid model displayed altered proliferation kinetics and growth deficits consistent with the clinical neuroimaging findings. Therefore, this cerebellar organoid model now allows the study of disease mechanisms underlying PCH2a for the first time.

### Challenges/limitations of current protocols

Despite the remarkable progress achieved in recent years, the field of cerebellar organoids is still in its early days, and the potential of these models is not yet fully realized. The main challenges include the lack of maturity, missing structural organization and heterogeneity of the developed models. Similarly to other brain organoids, cerebellar organoids represent an immature state of the developing brain (Nayler et al., 2021). Benchmarking of single-cell sequencing data obtained from cerebellar organoids against available data from the developing human cerebellum suggests that the cells in 1- to 3-month-old cerebellar organoids are roughly equivalent to cells in the developing human cerebellum during early- to mid-gestation (Atamian et al., 2024; Nayler et al., 2021; Sarieva et al., 2024 preprint).

Purkinje cells are the only output cell type of the cerebellar cortex, develop the earliest and show a characteristic highly complex morphology (Busch and Hansel, 2023). Consistent with the species-specific developmental timeline of cerebellar organoids, evident in transcriptomic data, Purkinje cell morphology in 6-month-old cerebellar organoids (assessed with immunostaining against CALB1) was suggested to resemble that of a human Purkinje cell in foetal tissue between post-conception weeks (PCW) 22 and 28 (Atamian et al., 2024). A more mature Purkinje cell morphology, including complex dendritic branching, was observed upon co-culture of human stem cell-derived Purkinje cells with dissociated mouse cerebellar neurons (Muguruma et al., 2015). These studies suggest that some of the cues that are required for neuronal maturation *in vivo* are currently lacking in the *in vitro* culture systems.

Microglia, brain-resident immune cells, are not normally part of current cerebellar organoid protocols and likely significantly affect cerebellar development (Stoessel and Majewska, 2021). In addition, neuronal activity by sensory inputs is absent *in vitro*, preventing activity-dependent neuronal maturation (Busch and Hansel, 2023). Finally, vascularization is missing, and interactions with the vasculature are important for normal brain development, for instance, to guide neuronal migration (Ruiz de Almodovar et al., 2010). The importance of *in vivo* conditions for the maturation of cerebellar Purkinje cells – including microglia, electrical activity and vasculature – has been demonstrated by enhanced morphological maturation upon transplantation of cerebellar organoids into the rat cerebellum (Amin et al., 2024).

Moreover, as mentioned above, only limited laminar layering has been achieved in cerebellar organoids to date (Atamian et al., 2024), which likely affects the formation of an organized cerebellar circuitry (Fig. 1C). Current organoid models do not model the characteristic foliated structure of the cerebellum, likely owing to the absence of scaling mechanisms and anchoring centres that usually drive cerebellar morphogenesis *in vivo* (Sudarov and Joyner, 2007). The relative immaturity of the generated cerebellar organoids, along with the other limitations listed above, needs to be taken into account when using this model system for the modelling of specific diseases, especially where the disease affects more mature neurons and networks.

## Implementing cerebellar organoids in the laboratory

### Ensuring a good start: choosing the right iPSC line

A key step in experimental design is choosing the appropriate starting cell line. Significant variability exists between different iPSC lines in terms of their differentiation potential, which poses challenges for reproducibility. A direct comparison of cerebellar organoids generated from different iPSC lines showed that resulting organoids can differ substantially in the generated cell populations and relative abundances of different cell types (Amin et al., 2024; Sarieva et al., 2024 preprint). This variability may arise from differences in cell type of origin, donor, culture conditions and reprogramming methods used to create iPSC lines (Carcamo-Orive et al., 2017), as well as different culture methods and differentiation protocols that are used in different laboratories (Volpato and Webber, 2020). Most variability seems to stem from genetic background, as assessed by transcriptomic (Carcamo-Orive et al., 2017; Scuderi et al., 2025) and proteomic (Beekhuis-Hoekstra et al., 2021) analysis of different iPSC lines. Variability between organoids poses challenges for reproducibility and disease modelling in different genetic contexts. As a consequence, the iPSC Neurodegenerative Disease Initiative (iNDI) has recently undertaken efforts to establish a reference iPSC line and introduce disease-causing variants (Pantazis et al., 2022). However, such an approach is not without flaws, as the reference line may be compromised by structural variants (Gracia-Diaz et al., 2024), although it is unclear how these changes will affect neurodevelopmental disease modelling (Ryan et al., 2024). Therefore, it is currently recommended to use a number of iPSC lines in parallel to identify robust phenotypes. In the case of patient-derived lines, it is recommended to generate isogenic control lines using gene-editing approaches to reduce variability.

The WTC-11 cell line is a well-characterized, widely available and widely used iPSC line for differentiation, genome editing and disease modelling in other organoid models, and has also been demonstrated to generate cerebellar organoids (Tolonen et al., 2024). Effective cerebellar differentiation has also been achieved with the female line AH017-3 (Tolonen et al., 2024); the male lines PGP1, 11a and D2 (Atamian et al., 2025); and the female lines F002.1A.13 and iPSC6.2 (Silva et al., 2021). Further benchmarking studies are required to generate a consensus on community-standard lines for the field.

### Maintaining human iPSC cultures

The most critical step of organoid formation is culturing high-quality, well-controlled iPSC lines prior to beginning cerebellar organoid differentiation. Poorly maintained iPSC lines will not yield reproducible organoid differentiations. We refer to other papers dedicated to maintaining high standards in iPSC modelling (Ludwig et al., 2023; Paşca et al., 2025) and a recent paper aimed at optimizing neural differentiation (Sutcliffe et al., 2026). The main considerations include starting from a well-qualified cell bank and keeping iPSCs in culture until a maximum of ten passages (Ludwig et al., 2023). No cell differentiation should be observed during cell culture. iPSC cultures should appear healthy, with sharp borders and prominent nucleoli, with ∼80-90% confluency just before starting differentiation protocols. It is essential to ensure that control iPSC lines are free of chromosome aberrations, maintain pluripotency, and are regularly checked for authenticity [short tandem repeat (STR) allele profiles] and mycoplasma contamination.

### Choosing the differentiation protocol

To date, four protocols for cerebellar organoid differentiation have been published (Atamian et al., 2024; Muguruma et al., 2015; Nayler et al., 2021; Silva et al., 2020) and independently validated. All protocols begin with 3D aggregate formation in the presence of a cell death inhibitor (ROCK inhibitor Y-27562) and TGF-β receptor inhibitor (SB431542), promoting specification of the neural ectoderm fate (Fig. 2A). Each protocol differs slightly in its patterning, timing and culturing conditions to specify cerebellar cell fate (Table 1, Fig. 2A). A rigorous side-by-side comparison of all protocols across multiple iPSC lines to assess their performance both within the same iPSC line in a single laboratory and across different laboratories has not yet been undertaken. Therefore, no quantitative data exist to compare the protocols directly.

Until then, it is imperative that the organoid batches are rigorously quality controlled prior to performing functional experiments, to identify an organoid protocol that works best with the chosen iPSC lines and for the specific scientific question. Notably, even in the field of neocortical development, in which organoids were first established (Kadoshima et al., 2013; Lancaster et al., 2013), and in which many different laboratories are active, to date, no uniform protocol exists; instead, a variety of differentiation protocols are used by different laboratories (He et al., 2024). To ensure reproducibility and traceability, we advocate for transparent methods sections that clearly describe the iPSC lines used, their culture conditions, and any modifications or deviations from published protocols.

Although two-dimensional (2D) protocols are not covered in this Special Article, it is worth noting that they can be used to derive certain specific cell types of the developing cerebellum. For example, granule cell progenitors can be generated through directed differentiation from iPSCs in 2D culture (Behesti et al., 2021). Organoids may also be dissociated into 2D cultures after a certain time point, for example, for electrophysiological analysis (Madencioglu et al., 2022; Silva et al., 2020). In general, dissociated and cultured neurons are generally more accessible than those in cerebellar organoids. Therefore, when experiments require higher cellular maturity, direct access to individual cells (for example, for continuous microscopic readouts) or higher throughput (for example, for screening), a 2D system may be more appropriate than 3D differentiation approach.

## Quality control of cerebellar organoids

When establishing a cerebellar differentiation protocol, or when using new iPSC lines with an established differentiation protocol, it is essential to perform rigorous quality control (QC) (Fig. 2). Well-maintained laboratory records, including tracking of catalogue and lot numbers, help identify any variability in cerebellar differentiation outcomes across users and over time.

### Size and shape

In control cell lines, organoids should be roughly spherical in shape, with an area of ∼0.2-0.8 mm^2^ at day 21 of differentiation and 0.8-3.14 mm^2^ at day 35 of differentiation (Fig. 2B). However, the size and shape of organoids can vary across different iPSC lines and may be part of a perturbation phenotype, as demonstrated in studies of PCH as described above (Kagermeier et al., 2024). Therefore, when working with less common iPSC lines, steady growth over time is more important than the specific size and shape mentioned above. When observing organoids under the phase-contrast microscope, bright, well-defined edges should be visible from day 7 onward throughout the differentiation process (Fig. 2B). In addition, organoids should not have blebs, protrusions, lobular structures, hollow cores or fall apart upon gentle movement by day 35 of differentiation (Boerstler et al., 2025). Organoid size should be tracked using wide-field microscopy to determine differences between control and experimental batches of organoids.

### Uniformity

Organoids from one batch and from the same iPSC line should be roughly similar in gene expression, protein markers, size and shape. Uniformity is best achieved by ensuring that the same seeding cell density per well is used during 3D aggregate formation at day 0. Each batch may have a few organoids that are inconsistent, especially organoids cultivated at the edges of 96-well plates. Wells located around the edges of the plate tend to lose moisture more quickly than those in the centre. As a result, those organoids have altered growth conditions and tend to be smaller. AggreWell plates (Stem Cell Technologies) may help produce aggregates of consistent sizes, as the plates are designed to force the aggregation of a defined number of cells (Antonchuk, 2013). However, phenotypic variation is also a common feature of *in vitro* differentiation. Mechanisms underlying the emergence of a broad phenotypic space are currently being investigated (Villaronga-Luque et al., 2025).

### Gene expression of cerebellar markers

Quantitative PCR (qPCR) is a rapid and accessible method to test the expression of candidate genes throughout organoid differentiation, enabling QC (batch consistency), validation of differentiation and commitment to the cerebellar fate (Table 2). To yield sufficient RNA, typically, 30-40 organoids are needed at day 21; 30 organoids at day 35; 10-15 organoids at day 50; and 5-10 organoids at days 70 and 90 (van Essen et al., 2024). By day 21 of differentiation, cerebellar organoids should express genes marking the hindbrain fate and lack genes marking the forebrain fate. Similarly, stem cell markers should also be downregulated. By day 35 of differentiation, specific cell types of the cerebellum should be detectable through gene expression analysis (Table 2; Table S1). By day 60, neurons and relative progenitors should have further matured, so organoids should express markers of more mature cell types.

**Table 2.** Quality control genes tested with quantitative PCR

Region/cell type	Marker genes	Expression relative to iPSCs	Timepoint
Hindbrain	*GBX2*	Upregulated	Day 21
	*EN1*	Upregulated	Day 21
	*EN2*	Upregulated	Day 21
Forebrain	*FOXG1*	Not detected	Day 21
	*OTX2*	Not detected	Day 21
iPSC	*OCT4*	Downregulated	Day 21
	*NANOG*	Downregulated	Day 21
	*ATOH1*	Upregulated	Day 35
RL progenitors	*LMX1A*	Upregulated	Day 35
	*PAX6*	Upregulated	Day 35
	*OLIG2*	Upregulated	Day 35
VZ progenitors	*PTF1A*	Upregulated	Day 35
	*KIRREL2*	Upregulated	Day 35
Glutamatergic lineage	*BARHL1*	Upregulated	Day 35
Early Purkinje cells	*LHX5*	Upregulated	Day 35
	*SKOR2*	Upregulated	Day 35
Neuronal and astrocyte marker	*TUBB3*	Upregulated	Day 60
Mature neuron marker	*RBFOX3*	Upregulated	Day 60
Purkinje cells	*CALB1*	Upregulated	Day 60
	*FOXP2*	Upregulated	Day 60
Granule neurons	*NEUROD1*	Upregulated	Day 60
	*TBR1*	Upregulated	Day 60
Cerebellar nuclei	*LHX9*	Upregulated	Day 60
	*SMI32*	Upregulated	Day 60
RL-SVZ/UBCs	*TBR2*	Upregulated	Day 60

iPSC, induced pluripotent stem cell; RL, rhombic lip; SVZ, subventricular zone; UBC, unipolar brush cell; VZ, ventricular zone.

### Protein expression of cerebellar markers

Other aspects of organoid QC, such as visualization of the spatial distribution and expression of specific cell types, can be assessed by immunofluorescence (IF) on sections or whole mounts of cryopreserved organoids. This method allows assessment of the relative abundance and spatial arrangement of cell types within an organoid, which is not possible through qPCR. Additionally, fewer organoids are needed for this analysis (*n*=3-5). By day 21 of differentiation, the organoids should develop polarized neuroepithelial tissue, characterized by flat-oval neural rosette-like structures (Muguruma et al., 2015). This polarity can also be observed with immunostaining for KIRREL2 and NCAD (also known as CDH2) markers (Muguruma et al., 2015). By day 35 of differentiation, all major cell types, including early Purkinje cells and RL progenitors, should be present (Table S2). Day 35 is a reliable time point for comparing organoids derived from different iPSC lines, as demonstrated in a recent preprint (Sarieva et al., 2024 preprint). Depending on the scientific question, organoids can be screened to identify which iPSC line generates organoids enriched for the specific cell types of interest, while ensuring the presence of all major cell types. The critical point is that the organoids exhibit a hindbrain identity and do not express forebrain markers throughout differentiation, as the protein markers for cerebellar cell types are also expressed in other brain regions (Atamian et al., 2025). By day 60, neuronal maturation can be determined using antibodies against TUJ1 (also known as TUBB3) and NeuN (also known as RBFOX3), and those against other cell- and stage-specific markers specified in Table S2.

## Experimental techniques for downstream analysis

In this section, we discuss different approaches that can be used for phenotypic readouts of cerebellar organoids following genetic or environmental perturbations, including published examples where available. For genetic perturbations, control and experimental organoids should ideally be isogenic, meaning that they differ only in the pathogenic variant but otherwise have an identical genetic background. This can be achieved by introducing the pathogenic variant into control iPSCs or by correcting the pathogenic variant in patient-derived iPSCs through CRISPR-based genome editing. All experiments should use more than one pair of control and experimental lines. Finally, iPSC lines and the cerebellar organoids derived from them should undergo standard QC as mentioned above, including verification of iPSC pluripotency and assessment of differentiation efficiency into cerebellar organoids.

### Introduction of disease-associated genes

The optimal timing of gene introduction depends on the specific disease being modelled and the putative effect of those genes on organoid formation. Examining hereditary diseases is likely best modelled by mutations introduced at the iPSC stage prior to differentiation, using CRISPR/Cas9 (for example, Martin et al., 2019; van Essen et al., 2024). Alternatively, if constitutive activation of disease variants interferes with organoid formation, the genes should be introduced after organoid differentiation, for example, using the PiggyBac transposon system delivered via electroporation (Ballabio et al., 2020; Lago et al., 2023). Disease genes can also be introduced by viral transduction [lentivirus, adeno-associated viruses (AAVs)] (Fischer et al., 2019). Cell type-specific expression of oncogenes can also be achieved by the introduction of plasmids encoding either Cre (Ballabio et al., 2021) or the reverse tetracycline-controlled transactivator under cell type-specific promoters as described in a recent preprint (Willott et al., 2024 preprint). The expression of genes is then controlled to specific cell types and time points by introducing an inducible cassette using a second plasmid (Ballabio et al., 2021).

In all cases, it is advisable to include a fluorescence protein that can be used to track the cells that successfully integrated the introduced genetic changes. QC measures include monitoring protein expression of the introduced transgene, either through western blotting or IF of cryopreserved organoids.

### Endogenous and transgenic reporters

To identify differences in specific cell types or cellular processes, fluorescent reporter iPSC lines can be used. Endogenous labelling enables real-time observation of specific cell types, or, in the case of protein labelling, subcellular processes. Reporter lines can be generated by editing the endogenous locus, which allows examination of gene expression and protein characteristics under native control elements, but takes longer to produce. Alternatively, fluorescent transgenes driven by the expression of promoter elements of a gene of interest can be generated relatively quickly, but their expression pattern may not reflect what occurs usually during development. For example, an iPSC reporter was developed recently to track endogenous gene expression of forkhead box P2 (*FOXP2*), a marker of early human Purkinje cells (Apsley et al., 2025). *FOXP2-*sorted neurons expressed a high number of genes associated with neurodevelopmental disease, including autism spectrum disorder, highlighting the possibility of using endogenously labelled reporter iPSC lines for disease modelling (Apsley et al., 2025). In addition, the Allen Institute for Cell Science offers fluorescently tagged iPSC lines derived from the WTC-11 parental line, which allow for imaging of 44 key cellular structures and substructures, such as the cytoskeleton, mitochondria and nuclear envelope (https://www.allencell.org/cell-catalog.html). Alternatively, reporters can be introduced via viral transduction or electroporation (Lago et al., 2023). For example, the introduction of a calcium reporter via AAV8 transduction allowed for the recording of cerebellar neuron intracellular calcium dynamics (Atamian et al., 2024).

### General comparisons using methods similar to QC

After introducing disease-associated or reporter genes, a practical starting point is to perform experiments similar to those described in the ‘Quality control of cerebellar organoids’ section. To minimize variability, differentiation of control and experimental organoids should begin on the same day. Shape and size serve as a first visual confirmation of any differences. For example, organoids with homozygous mutations in the SHH receptor *PTCH1* are larger and have a more lobular shape than control organoids at day 35 of differentiation (van Essen et al., 2024). qPCR can also be used at an early stage (day 21) to determine whether mutations of interest alter hindbrain patterning. IF can be used to monitor changes in abundance, subcellular localization and expression of proteins. To avoid artefacts, however, it also requires unbiased quantification for biological readouts (Schroder et al., 2024). Current challenges specific to brain organoids include organoid-to-organoid variability, section-to-section variability and the variability between batches. Therefore, to ensure robust and reliable results, examining at least four sections from at least three organoids (a total of 12 samples) in each condition over at least three independent rounds of differentiation is required (Schroder et al., 2024). In addition to IF, flow cytometry of a pool of live or fixed dissociated organoids can be used to measure the proportion of individual cell types within the organoids, mitigating the organoid-to-organoid and section-to-section variability (Silva et al., 2020). This approach is useful for examining a larger number of cells quickly in a more quantitative manner, but the spatial resolution is lost.

### Genome-wide transcriptomic analysis

In addition to qPCR to examine specific target genes, cerebellar organoids can also be pooled for transcriptomic analysis. This technique allows all genes to be analysed rather than picking a few genes in qPCR, but is more costly in both time and money. Using bulk RNA sequencing, one can examine gene transcription differences between control versus experimental organoids (Silva et al., 2021). Replicates are key for robust experiments. One should choose a strategy that combines technical replicates (several organoid pools from the same differentiation), differentiation replicates (several batches of differentiation) and true biological replicates (several cell lines, see discussion above).

The disadvantage of bulk RNA sequencing is that cellular diversity and heterogeneity are masked. Given the intrinsic cellular diversity of organoids, it is also advisable to perform scRNA-seq, in line with large-scale scRNA-seq efforts for other types of brain organoids (He et al., 2024). However, scRNA-seq experiments are costly, thereby requiring compromises in experimental design. scRNA-seq of cerebellar organoids has shown that they contain relevant cell types, such as the granule cell lineage and the Purkinje cell lineage (Atamian et al., 2024; Nayler et al., 2021). This method can also be used to identify differences in the abundance of specific genes and in gene expression across specific cell types under experimental treatment conditions (including disease-causing genetic variants) versus control organoids. scRNA-seq can be performed on single organoids or on pools of several organoids. Sequencing single organoids is ideal for understanding individual variability between experimental and control organoids, and it may also allow for easier identification of rare cell types. By sequencing pools of organoids, the data are averaged, which may mask heterogeneity but likely allows for a better assessment of the overall cellular composition and responses across the population.

Single-cell technology from the company 10x Genomics has been the preferred method in published papers (Atamian et al., 2024; Nayler et al., 2021), likely as the technology was widely adopted early in atlasing studies (for example, Zeisel et al., 2018). The 10x protocol uses a droplet-based method, in which individual cells or nuclei are captured in a droplet using microfluidics. However, a recent preprint has also benchmarked the newer single-cell technology in cerebellar organoids called the split-barpooling method, commercialized by Parse Biosciences (Sarieva et al., 2024 preprint), which may be a less expensive alternative. Parse technology uses a multiplexing, split-pool ligation-based technology, which uses combinatorial barcoding to index and fix cells in parallel, without physically separating them. Both technologies had a high technical reproducibility and similar cell type abundance calculations, which aligns with other published work (Xie et al., 2024); however, 10x processed samples tended to display higher levels of cell stress genes (Sarieva et al., 2024 preprint). The preprint showed that Parse required a higher starting cell number to capture a similar number of cells and revealed more genes of a greater length (>100 kb, so-called long genes) (Sarieva et al., 2024 preprint). Long genes are important for neural development and essential for cell adhesion, axon guidance and synapse formation (Gabel et al., 2015; Sugino et al., 2014), but they are also more vulnerable to DNA breaks and mutations (Wei et al., 2016), which may contribute to neurodevelopmental disorders.

### 3D imaging of organoids

In addition to imaging of 2D sections, it is also possible to perform staining on whole-mount samples, eliminating section-to-section variability. For this technique, the organoids should be cleared for 3D imaging (Eliat et al., 2022). Clearing is a process by which the optical properties of a tissue are changed so that light can better penetrate through the tissue, allowing for a greater depth of imaging (Weiss et al., 2021). Many tissue clearing methods exist (for review, see Vieites-Prado and Renier, 2021), and general protocols for brain organoids are available (Dekkers et al., 2019; Eliat et al., 2022). Following optical clearance and IF, whole organoids can be imaged by confocal or light-sheet microscopes. Confocal microscopes are likely to be the best choice when higher resolution is required, although the sample image field is typically smaller than that with light-sheet microscopes (Vieites-Prado and Renier, 2021). Light-sheet microscopes enable imaging of a larger volume in a shorter amount of time but require higher transparencies (Vieites-Prado and Renier, 2021). This method has been used recently to track neural organoids through their development using several live markers (Jain et al., 2025). Although 3D imaging requires specialized equipment and large computational power, it may give a better estimation of true cell number in control versus disease/experimental organoids than 2D imaging, circumventing some of the associated variability with 2D imaging of sectioned organoids. Additionally, experimental conditions affecting the 3D structure of the organoids may become more apparent in this setup.

### Measuring neuronal activity

The output of the brain is predominantly its electrical activity, which allows fast signalling in complex interconnected neural networks to drive different behaviours. Neuronal activity is established during prenatal human brain development: as the first neurons are being generated, the first synapses are being established (Molnár et al., 2020). Additionally, neurotransmitter-based signalling likely already occurs at the level of neural progenitor cells (Mayer et al., 2019). Therefore, the development of electrical activity within single neurons and the coordinated activity within neuronal networks within organoids is an important measure of their utility in modelling brain physiology. Because many neurological disorders affect neuronal activity, measuring neuronal activity is also important for disease modelling. Measuring neural activity in brain organoids is complicated owing to their 3D structure, limiting access to the inner parts of organoids at cellular resolution (for review, see Cha et al., 2025). Conventional electrophysiological measurements in brain organoids can be performed by (1) patch-clamp recordings, directly measuring membrane potential changes in individual cells; (2) calcium imaging, using changes in intracellular calcium detected through changes in fluorescence of calcium indicators; or (3) microelectrode arrays (MEAs) (Cha et al., 2025).

Current cerebellar organoid differentiation protocols yield active neurons as determined by these techniques. Atamian and colleagues performed calcium imaging using a genetically encoded calcium indicator delivered through an AAV (pAAV-CAG-SomaGCaMP6f2) to measure spontaneous activity (Atamian et al., 2025). They found higher activity in 6-month-old organoids than in the 2-month-old organoids. They also describe increased activity in Purkinje cells after optogenetic stimulation (Atamian et al., 2025). Finally, they validated active neurons by patch-clamp recordings on whole organoids, focusing on Purkinje neurons (PCP2/L7^+^ neurons labelled through viral transduction), a proportion of which displayed spontaneous repetitive firing reminiscent of *in vivo* electrophysiological activity of Purkinje cells (Atamian et al., 2024, 2025). By contrast, Chen et al. (2023) employed the third conventional electrophysiology method and measured electrical activity in cerebellar organoids using MEAs. They found that electrical activity increased as organoids matured from day 176 of differentiation to day 232. However, they report high variability between organoids that needs to be further analysed with more organoids recorded (Chen et al., 2023).

### Co-culture/assembloids

Although co-culture of cerebellar organoids with organoids of other brain regions has not yet been demonstrated, such assembloids hold great promise for understanding how cells from different brain regions interact. For example, the cerebellum projects to the neocortex through the thalamus and may thus coordinate information processing in the thalamus (McAfee et al., 2022). Better understanding how the cerebellum connects to the neocortex at the cellular and molecular level may also be beneficial in understanding the role of the cerebellum in autism spectrum disorder (Khan et al., 2015) and other neurodevelopmental disorders. Co-culture with other non-neuronal cell types, such as vascular cells or immune cells, may allow for better modelling of diseases associated with defects in these cell types, such as inflammation. In addition, co-culturing normal cerebellar organoids with tumour cells may provide an essential component of the microenvironment, to improve the fidelity of the cancer cell line to the original patient tumour, thereby improving preclinical models for these diseases. For example, co-culture of cerebellar organoids with medulloblastoma tumour cell lines has demonstrated that the gene expression profiles of the tumour cells change to better resemble patient samples, both in terms of heterogeneity and malignant cell states (van Essen et al., 2025).

## Conclusions

Cerebellar organoids represent an exciting model system for human development and pathologies research because they resemble key cerebellar developmental stages and cell types in a relevant genome context. Historically, the cerebellum has been an understudied brain region; however, recent work has demonstrated a fundamental role of the cerebellum in many motor and non-motor behaviours and their associated diseases (for review, see Guell and Schmahmann, 2020; Kim et al., 2024). These include both acquired and inherited diseases, such as cerebellar ataxias; neurodevelopmental conditions like cerebral palsy (Iwhiwhu et al., 2026); neurodegenerative diseases, such as Alzheimer's disease (Samstag et al., 2025); and, increasingly, psychiatric conditions, including autism spectrum disorder (Whitney et al., 2009). Cerebellar organoids could fill a key, critical role in better understanding the effect of mutations and environmental insults to the cerebellum in these and other relevant diseases. In addition, cerebellar organoids hold great promise for modelling specific aspects of human cerebellar development, including the compartmentalized structures of progenitor zones and specific Purkinje cell subtypes. Although this field is still in its infancy, recent publications and conferences (Kutscher et al., 2025) have demonstrated the potential of cerebellar organoids in understanding normal development and disease modelling. As new laboratories implement these technologies, it is absolutely essential to adopt high-standard rigorous protocols to ensure reproducibility of the findings, both within one research group and between different research groups. Therefore, due consideration should be given to study design, which our article can assist with. In addition, methods sections should be detailed and transparent, with any modifications or deviations from published protocols clearly documented. We hope that this Special Article and other recently published frameworks (Paşca et al., 2025) will help to ensure reliability and reproducibility of work in the field, and advance the use of cerebellar organoids for developmental and disease modelling.

## Supplementary Material

10.1242/dmm.052478_sup1 Supplementary information
