# Supplementary material for: Generating cerebellar organoids from pluripotent stem cells
Source: Dis Model Mech. 2026 Jan 30;19(1):dmm052478. doi: 10.1242/dmm.052478 (PMC12893041; doi:10.1242/dmm.052478)
Supplement: Supplementary information [file dmm-19-052478-s1.pdf]

Table S1. qPCR primers

Gene	Name	Sequences	Marker for	Reference
ACTB	*ACTB-f*	CAATGTGGCCGAGGACTTTG	Reference gene	(Muguruma et al., 2015)
	ACTB-r	CATTCTCCTTAGAGAGAAGTGG		
GAPDH	*GAPDH-f*	GTCTCCTCTGACTTCAACAGCG	Reference gene	(Du et al., 2015)
	GAPDH-r	ACCACCCTGTTGCTGTAGCCAA		
TBP	*TBP-f*	GCAAGGGTTTCTGGTTTGCC	Reference gene	(Augustyniak et al., 2019)
	TBP-r	CAAGCCCTGAGCGTAAGGTG		
ATOH1	*ATOH1-f*	CGCAATGTTATCCCGTCGTT	GCP, rhombic lip	Frederik Arnskötter
	ATOH1-r	GTTTGTAGCAGCTCGGACAA		
OTX2	*OTX2-f*	AACCTCCCATGAGGCTGTAA	Forebrain, Midbrain	Universal Probe Library; (Tailor et al., 2013)
	OTX2-r	GGTGGACAGGTTCAGAGTCC		
FOXG1	*FOXG1-f*	ATGATCCCCAAGTCCTCGTT	Forebrain	Universal Probe Library
	FOXG1-r	GTGGTGGTTGTCGTTCTGG		
PAX6	*PAX6-f*	GAATCAGAGAAGACAGGCCA	Neural lineage	(Silva et al., 2021)
	PAX6-r	GTGTAGGTATCATAACTCCG		
EN2	*EN2-f*	GGCGTGGGTCTACTGTACG	Midbrain/Hindbrain	(Behesti et al., 2021)
	EN2-r	TACCTGTTGGTCTGGAACTCG		
TBR1	*TBR1-f*	ATTTCACCGGCACCAAAC	Cerebellar nuclei (CN)	Universal Probe Library
	TBR1-r	GGGATCGAGACCAGAAATGTT		
TBR2	*TBR2-f*	ACCGCCACCAAACTGAGA	Unipolar brush cells (UBC)	(Shimizu et al., 2019)
	TBR2-r	AAGCTCAAGAAAGGAAACATGC		
ZIC1	*ZIC1-f*	ATCCACAAAAGGACGCACAC	GCP	Universal Probe Library
	ZIC1-r	GTCACAGCCCTCAAACTCG		
LMX1A	*LMX1A-f*	CTCCGCAACTTCTCTCTGCT	Early cerebellum, rhombic lip	Universal Probe Library
	LMX1A-r	TTAGGCCGTCCAGCATGT		
PAX2	*PAX2-f*	CACTGGCCAGGGAAGCTA	Hindbrain	Universal Probe Library
	PAX2-r	TGTACTGGGGGTGGCTGTA		

GBX2	*GBX2-f*	AAAGAGGGCTCGCTGCTC	Hindbrain	(Nayler et al., 2021)
	GBX2-r	GGTCGTCTTCCACCTTTGAC		
MEIS1	*MEIS1-f*	GCATGAATATGGGCATGGA	GNPs	Universal Probe Library
	MEIS1-r	CATACTCCCCTGGCATACTTTG		
GLI2	*GLI2-f*	CATGGAGCACTACCTCCGTTC	SHH pathway	(Zhang et al., 2022)
	GLI2-r	CGAGGGTCATCTGGTGGTAAT		
TAG1/CNTN2	*TAG1-f*	TCGGGCATGTACCAGTGTG	migrating GCPs	Frederik Arnskötter
	TAG1-r	GACGCCTCACGGGATTCAG		
CCND1	*CCND1-f*	CAATGACCCCGCACGATTTC	SHH pathway	(Shu et al., 2016)
	CCND1-r	CATGGAGGGCGGATTGGAA		
CCND2	*CCND2-f*	ACCTTCCGCAGTGCTCCTA	GCP, SHH pathway	(Qinyu et al., 2013)
	CCND2-r	CCCAGCCAAGAAACGGTCC		
MYCN	*MYCN-f*	CACAAGGCCCTCAGTACCTC	SHH pathway	(Jacobs et al., 2009)
	MYCN-r	ACCACGTCGATTTCTTCCTC		
GLI1	*GLI1-f*	GTGAGCCTGAATCTGTGTATG	SHH pathway	Frederik Arnskötter
	GLI1-r	ATGTGCTCGCTGTTGATG		
SKOR2	*SKOR2-f*	CCAGGTGTTAAAAGGAAACACA	Purkinje cell progenitor	(Muguruma et al., 2015)
	SKOR2-r	GCTCCCTTTTCATCTGATCCT		
KIRREL2	*KIRREL2-f*	CCTGAAGAAGAGGAGACAGGC	Purkinje cell progenitor	(van Essen et al., 2024)
	KIRREL2-r	TCCTCCAGAACCAGATCACTG		

Table S2. Published antibodies for IF

Antibody	Host	Company	Catalog #	Comments / Use	Specification / Localization	Reference
ALDOC	Mouse	Santa Cruz		IF 1:200	aldolase C; Purkinje cells	(Muguruma et al., 2015; Silva et al., 2020)
ATOH1	Rabbit	Proteintech	21215-1-AP	IF 1:600	RL, GCPs	Frederik Arnskötter
ATOH1	Rabbit	Millipore		IF 1:200	RL, GCPs	(Watson et al., 2018)
ATOH1	Rabbit	Abclonal	A11477	IF 1:200	RL, GCPs	(Atamian et al., 2024)
BARHL1	Rabbit	Atlas Antibodies	HPA004809	IF 1:500; 1:1000 (B)	migrating GCPs	(Atamian et al., 2024; Muguruma et al., 2015; Silva et al., 2020)
CALB1	Mouse	Swant	300	IF 1:1000	Purkinje cells	(Muguruma, 2017)
CALB1	Rabbit	Swant		IF 1:500 (S); 1:5000 (W)	Purkinje cells	(Silva et al., 2020; Watson et al., 2018)
CALB1	Guinea Pig	Synaptic Systems	214004	IF 1:300	Purkinje cells	(Muguruma et al., 2015)
CALB1	Mouse	Sigma-Aldrich	C9848	IF 1:300	Purkinje cells	(Atamian et al., 2024)
CBLN1	Rabbit	Abcam		IF 1:200	Purkinje cells	(Muguruma et al., 2015)
CORL2	Rabbit	Atlas Antibodies		IF 1:100	Postmitotic Purkinje cell precursor (PCP); after cell cycle exit	(Silva et al., 2020)
EN1	Rabbit	Abcam	(ab70993)	IF 1:50	midbrain/hindbrain	(Nayler et al., 2021; Watson et al., 2018)
EN1	Rabbit	Millipore	ab5732	IF 1:200	midbrain/hindbrain	(Sagal et al., 2014)
EN2	Goat	Santa Cruz	sc-8111	IF 1:100 (S, M) 1:50 (W, B)	midbrain/hindbrain	(Behesti et al., 2021; Muguruma, 2017; Silva et al., 2020; Watson et al., 2018)
FOXA2	Rabbit	Cell Signaling		IF 1:200	dopaminergic neurons	(Sagal et al., 2014)
FOXG1	Rabbit	Abcam	ab18259	IF 1:200	forebrain neurons	(Atamian et al., 2024)
FOXP2	Goat	Abcam		IF 1:5000	FOXP2+ Purkinje cells	(Watson et al., 2018)
GAD65	Mouse	BD Pharmigen	559931	IF 1:200	Mature GABAergic neurons; glutamic acid decarboxylase isoform 65; presynaptic boutons	(Muguruma, 2017)
GBX2	Goat	Santa Cruz		IF 1:50	hindbrain	(Watson et al., 2018)
GBX2	Mouse	Abnova		IF 1:250	hindbrain	(Nayler et al., 2021)

Disease Models & Mechanisms • Supplementary information

GFAP	Chicken	Encor Biotech	CPCA-GFAP	IF 1:1500	glial cells	(Behesti et al., 2021)
GLURD2	Goat	Santa Cruz	sc-26118	IF 1:100	glutamate receptor	(Muguruma, 2017)
KI67	Mouse	BD Pharmigen	550609	IF 1:200 (Muguruma 2015)	cell cycle	(Muguruma et al., 2015)
KIRREL2/ NEPH3	Goat	R&D	AF2930	IF 1:500	Purkinje cells	(Muguruma, 2017; Nayler et al., 2021)
KIRREL2/ NEPH3	Mouse	R&D	MAB2564	IF 1:50	Purkinje cells	(Muguruma, 2017)
KIRREL2/ NEPH3	Rabbit	Proteintech	10890-1-AP	IF 1:100	Purkinje cells	(Atamian et al., 2024)
L7/PCP2	Mouse	Santa Cruz		IF 1:200	Purkinje cell protein 2 (PCP2)	(Silva et al., 2020)
L7	Rabbit	Takara/Clonetech	M202	IF 1:500	Purkinje cell protein 2 (PCP2)	(Muguruma, 2017)
LMX1A	Goat	Santa Cruz	sc-54273	IF 1:100	RL	(Muguruma, 2017)
MAP2	Mouse	Sigma Aldrich	M1406	IF 1:1000 (Silva) 1:500 (Mu)	microtubles in dendrites of postmitotic neurons	(Muguruma et al., 2015; Silva et al., 2020)
MAP2	Chicken	Abcam	ab5392	1:1000	microtubles in dendrites of postmitotic neurons	(Behesti et al., 2021)
NCAD	Mouse	BD Transduction	610920	IF 1:1000	N-cadherin	(Muguruma et al., 2015; Silva et al., 2020)
NEUN	Mouse	Millipore	MAB377	IF 1:100 (B); 1:500 (K)	mature neurons	(Behesti et al., 2021)
NEUROD1	Mouse	BD Pharmigen	563000	IF 1:300	granule cells	(Behesti et al., 2021)
NRGN	Rabbit	Millipore		IF 1:200 (S) 1:1000 (M)	Neurogranin in cytoplasm; Golgi cell	(Muguruma et al., 2015; Silva et al., 2020)
OLIG2	Goat	R&D	AF2418	IF 1:100 (M) 1:40 (W)	Neurogenic precursor/nascent Purkinje cells (cell cycle exit & differentiation)	(Muguruma, 2017; Watson et al., 2018)
OLIG2	Rabbit	Millipore	AB9610	IF 1:500; 1:1000 (M)	Neurogenic precursor/nascent Purkinje cells (cell cycle exit & differentiation)	(Muguruma et al., 2015; Silva et al., 2020)
OTX2	Goat	Santa Cruz	sc-30659	IF 1:100	forebrain (early), posterior cerebellum (late)	(Muguruma et al., 2015)
OTX2	Rabbit	Abcam	ab21990	IF 1:100	forebrain (early), posterior cerebellum (late)	(Muguruma et al., 2015)
OTX2	Mouse	R&D	MAB1979	IF 1:1000	forebrain (early), posterior cerebellum (late)	(Muguruma et al., 2015)

Disease Models & Mechanisms • Supplementary information

PAX2	Mouse	Abnova		IF 1:400	GABAergic interneurons (IN) & precursors; Golgi cell	(Muguruma et al., 2015; Silva et al., 2020)
PAX2	Rabbit	Zymed		IF 1:200	glutamatergic cells	(Muguruma et al., 2015)
PAX6	Rabbit	Biolegend	901301	IF 1:300 (B)	glutamatergic cells	(Atamian et al., 2024; Behesti et al., 2021)
PAX6	Goat	Santa Cruz		IF 1:100	glutamatergic cells	(Muguruma et al., 2015)
PAX6	Rabbit	Covance		IF 1:400 (S) 1:500 (M)	glutamatergic cells	(Muguruma, 2017; Sagal et al., 2014; Silva et al., 2020)
PTCH1	Rabbit	abcam	ab53715	WB 1:1000	SHH pathway	(Susanto et al., 2020)
PTF1A	Mouse	Sigma Aldrich	WH0256297M5		VZ progenitors	Frederik Arnskötter
PVALB	Mouse	Sigma Aldrich		IF 1:200 (S) 1:1000 (M)	GABAergic interneurons	(Muguruma et al., 2015; Silva et al., 2020)
SIX3	Rabbit	abcam	ab221750	IF 1:250	Forebrain	(Walsh et al., 2020)
SKOR2	Rabbit	Atlas Antibodies	HPA046206	IF 1:100; 1:1000 (B)	Ventricular zone-derived postmitotic Purkinje cell progenitors	(Ballabio et al., 2020; Muguruma et al., 2015)
SOX2	Mouse	R&D		IF 1:200 (S) 1:100 (M)	stem cells	(Muguruma et al., 2015; Silva et al., 2020)
SOX2	Goat	Santa Cruz		IF 1:300	stem cells	(Muguruma et al., 2015)
SOX2	Goat	R&D	AF2018	IF 1:500	stem cells	(Atamian et al., 2024)
SYNAPTOPHYSIN	Mouse IgM	Millipore	MAB329	IF 1:500	synaptic vesicles	(Behesti et al., 2021)
TBR1	Rabbit	Millipore		IF 1:200	Cerebellar nuclei (CN)	(Silva et al., 2020)
TBR1	Rabbit	Abcam	ab31940	IF 1:1000 (M), 1:200 (A)	Cerebellar nuclei (CN)	(Atamian et al., 2024; Muguruma et al., 2015)
TBR1	Rabbit	Millipore		IF 1:1000	Cerebellar nuclei (CN)	(Muguruma et al., 2015)
TBR2	Rabbit	Abcam	ab23345	IF 1:200	Unipolar brush cells (UBC)	(Silva et al., 2020)
TTR	Rabbit	Proteintech	11891-1-AP	IF 1:100	choroid plexus	(Atamian et al., 2024)
TUJ1	Mouse	Biolegend		IF 1:1000	neurons and astrocytes	(Silva et al., 2020)
TUJ1	Mouse	Covance	MMS-435P	IF 1:500 (M); 1:1000 (W)	neurons and astrocytes	(Muguruma, 2017; Watson et al., 2018)
TUJ1	Rabbit	Covance	PRB-435P	IF 1:2000	neurons and astrocytes	(Muguruma et al., 2015)
VGLUT1	Mouse	Synaptic Systems		IF 1:100	glutamatergic marker	Frederik Arnskötter
ZO1	Rabbit	Novex		IF 1:100	Cytoplasmic side of tight junctions	(Silva et al., 2020)

Disease Models & Mechanisms • Supplementary information

References

Atamian, A., Birtele, M., Hosseini, N., Nguyen, T., Seth, A., Del Dosso, A., Paul, S., Tedeschi, N., Taylor, R., Coba, M.P., Samarasinghe, R., Lois, C., Quadrato, G., 2024. Human cerebellar organoids with functional Purkinje cells. Cell Stem Cell 31, 39-51.e6. https://doi.org/10.1016/j.stem.2023.11.013

Augustyniak, J., Lenart, J., Lipka, G., Stepien, P.P., Buzanska, L., 2019. Reference Gene Validation via RT–qPCR for Human iPSC-Derived Neural Stem Cells and Neural Progenitors. Mol Neurobiol 56, 6820–6832. https://doi.org/10.1007/s12035-019-1538-x

Ballabio, C., Anderle, M., Gianesello, M., Lago, C., Miele, E., Cardano, M., Aiello, G., Piazza, S., Caron, D., Gianno, F., Ciolfi, A., Pedace, L., Mastronuzzi, A., Tartaglia, M., Locatelli, F., Ferretti, E., Giangaspero, F., Tiberi, L., 2020. Modeling medulloblastoma in vivo and with human cerebellar organoids. Nat Commun 11, 583. https://doi.org/10.1038/s41467-019-13989-3

Behesti, H., Kocabas, A., Buchholz, D.E., Carroll, T.S., Hatten, M.E., 2021. Altered temporal sequence of transcriptional regulators in the generation of human cerebellar granule cells. eLife 10, e67074. https://doi.org/10.7554/eLife.67074

Du, C., Weng, X., Hu, W., Lv, Z., Xiao, H., Ding, C., Gyabaah, O.K., Xie, H., Zhou, L., Wu, J., Zheng, S., 2015. Hypoxia-inducible MiR-182 promotes angiogenesis by targeting RASA1 in hepatocellular carcinoma. J Exp Clin Cancer Res 34, 67. https://doi.org/10.1186/s13046-015-0182-1

Jacobs, J.F., Van Bokhoven, H., Van Leeuwen, F.N., Hulsbergen-van De Kaa, C.A., De Vries, I.J.M., Adema, G.J., Hoogerbrugge, P.M., De Brouwer, A.P., 2009. Regulation of MYCNexpression in human neuroblastoma cells. BMC Cancer 9, 239. https://doi.org/10.1186/1471-2407-9-239

Muguruma, K., 2017. 3D Culture for Self-Formation of the Cerebellum from Human Pluripotent Stem Cells Through Induction of the Isthmic Organizer, in: Tsuji, T. (Ed.), Organ Regeneration, Methods in Molecular Biology. Springer New York, New York, NY, pp. 31–41. https://doi.org/10.1007/978-1-4939-6949-4_3

Muguruma, K., Nishiyama, A., Kawakami, H., Hashimoto, K., Sasai, Y., 2015. Self-organization of polarized cerebellar tissue in 3D culture of human pluripotent stem cells. Cell Rep 10, 537–550. https://doi.org/10.1016/j.celrep.2014.12.051

Nayler, S., Agarwal, D., Curion, F., Bowden, R., Becker, E.B.E., 2021. High-resolution transcriptional landscape of xeno-free human induced pluripotent stem cell-derived cerebellar organoids. Sci Rep 11, 12959. https://doi.org/10.1038/s41598-021-91846-4

Qinyu, L., Long, C., Zhen-dong, D., Min-min, S., Wei-ze, W., Wei-ping, Y., Cheng-hong, P., 2013. FOXO6 promotes gastric cancer cell tumorigenicity via upregulation of C-myc. FEBS Letters 587, 2105–2111. https://doi.org/10.1016/j.febslet.2013.05.027

Sagal, J., Zhan, X., Xu, J., Tilghman, J., Karuppagounder, S.S., Chen, L., Dawson, V.L., Dawson, T.M., Laterra, J., Ying, M., 2014. Proneural Transcription Factor Atoh1 Drives Highly Efficient Differentiation of Human Pluripotent Stem Cells Into Dopaminergic Neurons. Stem Cells Translational Medicine 3, 888–898. https://doi.org/10.5966/sctm.2013-0213

Shimizu, K., Sato, Y., Kawamura, M., Nakazato, H., Watanabe, T., Ohara, O., Fujii, S., 2019. Eomes transcription factor is required for the development and differentiation of invariant NKT cells. Commun Biol 2, 150. https://doi.org/10.1038/s42003-019-0389-3

Shu, Q., Liu, J., Liu, X., Zhao, S., Li, H., Tan, Y., Xu, J., 2016. GABA$_B$ R / GSK -3 β / NF - κ B signaling pathway regulates the proliferation of colorectal cancer cells. Cancer Medicine 5, 1259–1267. https://doi.org/10.1002/cam4.686

Silva, T.P., Bekman, E.P., Fernandes, T.G., Vaz, S.H., Rodrigues, C.A.V., Diogo, M.M., Cabral, J.M.S., Carmo-Fonseca, M., 2020. Maturation of Human Pluripotent Stem Cell-Derived Cerebellar Neurons in the Absence of Co-culture. Front Bioeng Biotechnol 8, 70. https://doi.org/10.3389/fbioe.2020.00070

Silva, T.P., Sousa-Luís, R., Fernandes, T.G., Bekman, E.P., Rodrigues, C.A.V., Vaz, S.H., Moreira, L.M., Hashimura, Y., Jung, S., Lee, B., Carmo-Fonseca, M., Cabral, J.M.S., 2021. Transcriptome profiling of human pluripotent stem cell-derived cerebellar organoids reveals faster commitment under dynamic conditions. Biotechnol Bioeng 118, 2781–2803. https://doi.org/10.1002/bit.27797

Susanto, E., Navarro, A.M., Zhou, L., Sundström, A., Bree, N. van, Stantic, M., Moslem, M., Tailor, J., Rietdijk, J., Zubillaga, V., Hübner, J.M., Weishaupt, H., Wolfsberger, J., Alafuzoff, I., Nordgren, A., Magnaldo, T., Siesjö, P., Johnsen, J.I., Kool, M., Tammimies, K., Darabi, A., Swartling, F.J., Falk, A., Wilhelm, M., 2020. Modeling SHH-driven medulloblastoma with patient iPS cell-derived neural stem cells. Proceedings of the National Academy of Sciences of the United States of America 117, 20127–20138. https://doi.org/10.1073/PNAS.1920521117

Tailor, J., Kittappa, R., Leto, K., Gates, M., Borel, M., Paulsen, O., Spitzer, S., Karadottir, R.T., Rossi, F., Falk, A., Smith, A., 2013. Stem cells expanded from the human embryonic hindbrain stably retain regional specification and high neurogenic potency. J Neurosci 33, 12407–12422. https://doi.org/10.1523/JNEUROSCI.0130-13.2013

van Essen, M.J., Apsley, E.J., Riepsaame, J., Xu, R., Northcott, P.A., Cowley, S.A., Jacob, J., Becker, E.B.E., 2024. PTCH1-mutant human cerebellar organoids exhibit altered neural development and recapitulate early medulloblastoma tumorigenesis. Dis Model Mech 17, dmm050323. https://doi.org/10.1242/dmm.050323

Walsh, P., Truong, V., Nayak, S., Saldías Montivero, M., Low, W.C., Parr, A.M., Dutton, J.R., 2020. Accelerated differentiation of human pluripotent stem cells into neural lineages via an early intermediate ectoderm population. Stem Cells 38, 1400–1408. https://doi.org/10.1002/stem.3260

Watson, L.M., Wong, M.M.K., Vowles, J., Cowley, S.A., Becker, E.B.E., 2018. A Simplified Method for Generating Purkinje Cells from Human-Induced Pluripotent Stem Cells. Cerebellum 17, 419–427. https://doi.org/10.1007/s12311-017-0913-2

Zhang, Y., Yao, G., Yang, X., Qiu, T., Wang, S., 2022. Mechanism of Targeting the Hedgehog Signaling Pathway against Chemotherapeutic Resistance in Multiple Myeloma. Journal of Oncology 2022, 1–9. https://doi.org/10.1155/2022/1399697
